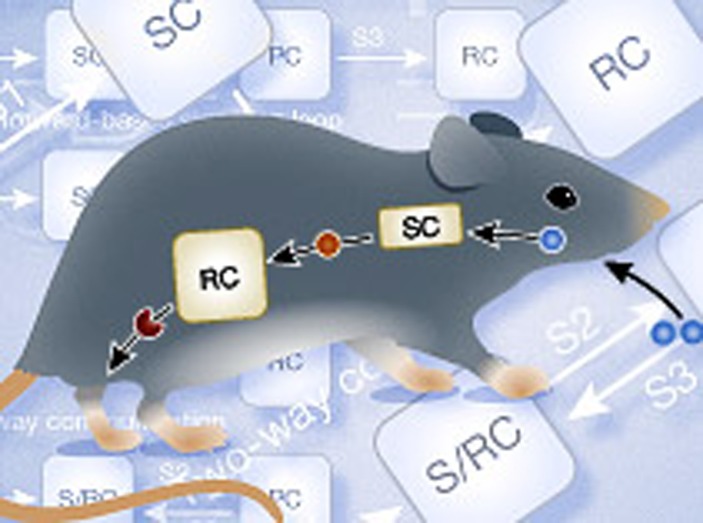# Biomedically relevant circuit-design strategies in mammalian synthetic biology

**DOI:** 10.1038/msb.2013.48

**Published:** 2013-09-24

**Authors:** William Bacchus, Dominique Aubel, Martin Fussenegger

**Affiliations:** 1 Department of Biosystems Science and Engineering, ETH Zurich, Basel, Switzerland; 2 IUTA Département Génie Biologique, Université Claude Bernard Lyon 1, Villeurbanne Cedex, France; 3 Faculty of Science, University of Basel, Basel, Switzerland

**Keywords:** gene circuits, mammalian designer devices, synthetic biology

## Abstract

The development and progress in synthetic biology has been remarkable. Although still in its infancy, synthetic biology has achieved much during the past decade. Improvements in genetic circuit design have increased the potential for clinical applicability of synthetic biology research. What began as simple transcriptional gene switches has rapidly developed into a variety of complex regulatory circuits based on the transcriptional, translational and post-translational regulation. Instead of compounds with potential pharmacologic side effects, the inducer molecules now used are metabolites of the human body and even members of native cell signaling pathways. In this review, we address recent progress in mammalian synthetic biology circuit design and focus on how novel designs push synthetic biology toward clinical implementation. Groundbreaking research on the implementation of optogenetics and intercellular communications is addressed, as particularly optogenetics provides unprecedented opportunities for clinical application. Along with an increase in synthetic network complexity, multicellular systems are now being used to provide a platform for next-generation circuit design.

## Introduction

Mammalian synthetic biology has established itself in only a few years as one of the strongest and most innovative biological disciplines (Grushkin, 2012). What began as simple transcriptional gene switches responding to supplied inducers has become an ever-expanding toolbox of genetically encoded circuits with highly complex functionality. The design of such mammalian circuits has proliferated, and is now able to apply regulatory mechanisms at the DNA, RNA or protein levels, or in some combination thereof (Keefe et al, 2010; Wieland and Fussenegger, 2012; Wang et al, 2013). The arsenal of circuits now available includes genetic toggle switches (Kramer et al, 2004b; Greber et al, 2008), band-pass filters (Greber and Fussenegger, 2010), time delay circuits (Weber et al, 2007b), memory devices (Burrill et al, 2012), oscillators (Tigges et al, 2009) and biocomputers (Benenson, 2011; Auslander et al, 2012a; Daniel et al, 2013). Circuits have been designed for diverse purposes, including to perform logic calculations (Rinaudo et al, 2007; Auslander et al, 2012a), screen for anti-tuberculosis compounds (Weber et al, 2008), control T-cell proliferation (Chen et al, 2010), kill cancer cells (Xie et al, 2011) or treat metabolic disorders (Kemmer et al, 2010; Ye et al, 2011, 2013).

However, despite increased complexity and highly innovative circuit design, synthetic biology's current state is still that of a ‘proof of concept' discipline. To progress toward clinically relevant applications, synthetic biology design has changed drastically in recent years. Of crucial importance are both the design of regulatory circuits and the biocompatibility of regulatory compounds. The original gene switches were constructed to respond to compounds with potential pharmacological side effects, such as antibiotics (Fussenegger et al, 2000; Weber et al, 2002). Newer circuits aim to reduce potentially negative impacts on patients, and therefore use food components and food additives such as vitamins and amino acids (Weber et al, 2007b; Bacchus et al, 2012), cell metabolites (Weber et al, 2007a; Wang et al, 2008), signaling transduction partners (Culler et al, 2010) and even endogenous cell type-specific transcription factors (Nissim and Bar-Ziv, 2010) to regulate the circuit function. This enables synthetic circuits to be directly integrated with the patient's metabolic networks to interface and respond to endogenous signals already present in the patient (Weber and Fussenegger, 2012).

Part of the success of mammalian synthetic biology has been due to its ability to constantly improve and create more advanced and robust genetic circuits. But another part of its success has been its ability to interact with other emerging biological disciplines (Ehrbar et al, 2008; Milias-Argeitis et al, 2011; Guo et al, 2012; Heng et al, 2013), the most obvious example being optogenetics (Chow et al, 2010; Chow and Boyden, 2011). Research at the interface with optogenetics has led to the development of non-invasive traceless methods of regulating various cellular functions by simple light irradiation (Levskaya et al, 2005; Tyszkiewicz and Muir, 2008; Yazawa et al, 2009; Kennedy et al, 2010; Ye et al, 2011; Wang et al, 2012; Bugaj et al, 2013; Muller et al, 2013a, 2013b). Photoreceptors, the sensory building blocks of optogenetic circuits, are abundant in nature. They are continually being identified, characterized and genetically modified by researchers, and therefore provide a constant flow of novel building blocks for constructing light-responsive synthetic biology tools (Airan et al, 2009; Chow et al, 2010).

Multicellular organisms consist of different consortia of specialized cells that have evolved to execute and coordinate, by intercellular communication, specific activities to distribute highly complex tasks and workload to increase the overall fitness of the organism. Likewise, with synthetic biology-based circuits becoming increasingly complex and multilayered (Auslander et al, 2012a; Moon et al, 2012), a single designer cell will no longer be able to cope with the complexity of programmed functionalities. To ovecome this limitation, engineered activities and metabolic workload will need to be distributed among different communicating designer cell populations that coordinate their activities to provide concerted actions. The design and construction of synthetic intercellular communication has thus far provided a ready and sustainable solution (Li and You, 2011). Engineering specialized and interconnected cell populations allows for a plug-and-play approach where the combinations themselves determine the overall function of the cellular consortium (Regot et al, 2011; Tamsir et al, 2011). Synthetic multicellular consortia of communicating cell populations show increased control precision and reliability (Koseska et al, 2009) and will foster advances in tissue engineering, the assembly of complex cellular patterns with novel functionalities (Liu et al, 2011), and the design of synthetic hormone systems (Weber et al, 2007a). Also, the distribution of synthetic circuits among specialized cell populations may overcome apparent limitations in the engineering capacity and metabolic activities of individual cells and will enable the design of increasingly complex multicellular gene networks (Bacchus et al, 2012; Rusk, 2012).

In this review, we cover the novel repertoire of mammalian synthetic circuit design. We discuss regulatory circuits that enable a direct link between synthetic biology and endogenous cellular activities, continuing advances in circuit design, synthetic circuits that implement optogenetic features, and conclude with a discussion of synthetic intercellular communication and prosthetic networks.

## Synthetic circuits based on rewired cell-signaling pathways

To integrate synthetic circuits with endogenous signaling pathways, cells are engineered to express transmembrane receptors that respond via endogenous signal transduction pathways. In this way, the circuits use the natural signaling mechanism of the cell to regulate cellular functions. This can be done in a direct way, via elevated levels of second messengers (Airan et al, 2009), or an indirect way, via activation of synthetic promoters (Kemmer et al, 2011; Ye et al, 2011, 2013; Stanley et al, 2012). This design enables a generic strategy for constructing synthetic control systems, which can be designed to respond to either endogenous or externally applied stimuli depending on which receptor is used.

**Figure 1:**
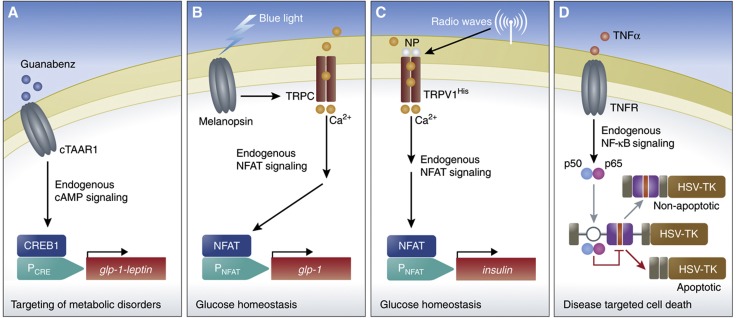
Synthetic circuits based on the rewired cell-signaling pathways. (**A**) Guanabenz-induced synthetic circuit for the treatment of metabolic syndrome. Cells engineered to express the chimeric trace amine-associated receptor (cTAAR1) respond to Guanabenz by activating endogenous cAMP signaling. Increased levels of cAMP activate P_CRE_-driven transgene expression of Glp-1-Leptin via a cAMP-responsive element binding protein 1 (CREB1). When implanted in mice developing symptoms of metabolic syndrome, the circuit enabled simultaneous targeting of several metabolic disorders (Ye et al, 2013). (**B**) Blue light- and (**C**) radio wave-induced synthetic circuits enabling glucose homeostasis. (**B**) Cells engineered to trigger calcium influx through transient receptor potential channels (TRPCs) by expressing blue light-responsive melanopsin, link blue-light sensing to transgene expression via an NFAT-responsive promoter (P_NFAT_). Implanted in diabetic mice, the circuit enabled blue light-controlled glucose homeostasis when expressing glucagon-like peptide 1 (Ye et al, 2011). (**C**) Cells engineered to trigger calcium influx through temperature-sensitive, His-tagged TRPCs (TRPV1^HIS^). Antibody-coated nanoparticles for His-tag recognition (NP) enabled local nanoparticle heating of TRPV1^HIS^, consequently allowing for calcium influx, linking radio-wave exposure to transgene expression via an NFAT-responsive promoter (P_NFAT_). Implanted in mice, the circuit enabled radio wave-controlled regulation of blood glucose levels by expressing insulin (Stanley et al, 2012). (**D**) Synthetic circuit responsive to endogenous proteins allow for disease-targeted cell death. The RNA-based devise is composed of specific aptamers for p50/p65 recognition (white circle), localized at key intronic positions near an alternative spliced exon harboring a stop codon (red area) in a three-exon, two-intron minigene fused to a suicide gene (HSV-TK). Activation of the NF-κB pathway by stimulation of the tumor necrosis factor receptor (TNFR) with tumor necrosis factor-α (TNFα) enables p50/p65 regulation of exon exclusion, thereby linking disease markers to the killing of the diseased cells (Culler et al, 2010).

This strategy was adopted to construct a synthetic circuit for the treatment of the metabolic syndrome, a collection of interdependent pathologies including hypertension, hyperglycemia, obesity and dyslipidemia. Cells were engineered to express a chimeric trace amine-associated receptor 1 (cTAAR1), which produced a stronger cAMP response compared with its native counterpart in response to the clinically licensed antihypertensive drug Guanabenz (Wytensin®) (Ye et al, 2013). Increased intercellular cAMP levels triggered transgene expression from a synthetic promoter (P_CRE_) via the cAMP-responsive element binding protein 1 (CREB1). In this way, the oral dose of Guanabenz was simultaneously controlling hypertension as well as expression of a bifunctional therapeutic peptide hormone, GLP-1-Leptin, which combines the anorexic and insulin secretion-stimulating effect of the glucagon-like peptide 1 (GLP-1) with the lipid level, food intake- and body weight-controlling capacity of leptin. Implanting the circuit in mice that were developing symptoms of the metabolic syndrome (*ob/ob* mice) enabled simultaneous correction of all associated pathologies (Figure 1A) (Ye et al, 2013).

Melanopsin, the photopigment of retinal ganglion cells that interacts with retinal (vitamin A), has been utilized to induce light sensitivity in otherwise non-sensitive cells (Melyan et al, 2005). In retinal ganglion cells, blue-light stimulation of melanopsin activates transient receptor potential channels (TRP channels) via a G-protein signaling cascade, resulting in calcium influx. By linking melanopsin signal transduction to the endogenous signaling pathway of the nuclear factor of activated T cells (NFAT), which is responsive to elevated calcium levels, Ye et al (2011) constructed a blue light-responsive circuit that enabled transgene expression from an NFAT-responsive promoter. Expression of the GLP-1 under the control of the NFAT-responsive promoter resulted in blue light-controlled blood-glucose homeostasis in type 2 diabetic mice (Figure 1B) (Ye et al, 2011).

In a similar manner, Stanley et al (2012) utilized the endogenous signaling pathway of NFAT to regulate gene expression directly by engineering the control of TRP channel activation, in an approach that combined synthetic biology with nanotechnology. Iron oxide nanoparticles coated with His antibodies were targeted to a temperature-sensitive TRP channel, which had been modified to express extracellular His-epitope tags (TRPV1^His^). The metal nanoparticles absorb radio-wave energy and transfer the heat to the temperature-sensitive TRPV1^His^, which opens the channel and triggers calcium influx. These elevated calcium levels resulted in transgene expression from an NFAT-responsive promoter, and when used in mice, radio wave-heated activation of a modified human insulin gene was able to regulate glucose levels in the animals (Figure 1C) (Stanley et al, 2012).

Culler et al (2010) reported a highly sophisticated strategy to use the recognition of disease markers to reprogram cell fate. They constructed an RNA-based device composed of specific aptamers designed to recognize endogenous signaling partners such as the subunits p50 and p65 of the transcription factor NF-κB. The aptamers were placed into key intronic locations near an alternatively spliced exon that harbored a stop codon. The exclusion of the alternative exon, which was part of a three-exon, two-intron minigene fused to a suicide gene (HSV-TK), was dependent on the binding of the p50 and p65 subunits to the aptamers. In the presence of tumor necrosis factor-α, the NF-κB pathway was induced, leading to the translocation of p50 and p65 to the nucleus. Subsequently, their presence in the nucleus regulated exon exclusion of the alternative exon and HSV-TK expression, ultimately resulting in cell death (Figure 1D) (Culler et al, 2010).

## Sophisticated two-/multi-input design allows for increased circuit complexity

The successful development of synthetic gene circuits mainly rests on the construction of gene regulation systems where one specific input is converted by the circuit into a specific genetic output. These circuits are likely to have limitations in therapeutic settings, as disease states typically have complex biological profiles (Evan and Littlewood, 1998; Banegas et al, 2007). Recent work in synthetic biology is therefore focused on constructing two-input or even multiple-input circuits where combinations of the input signals determine the final genetic response.

**Figure 2:**
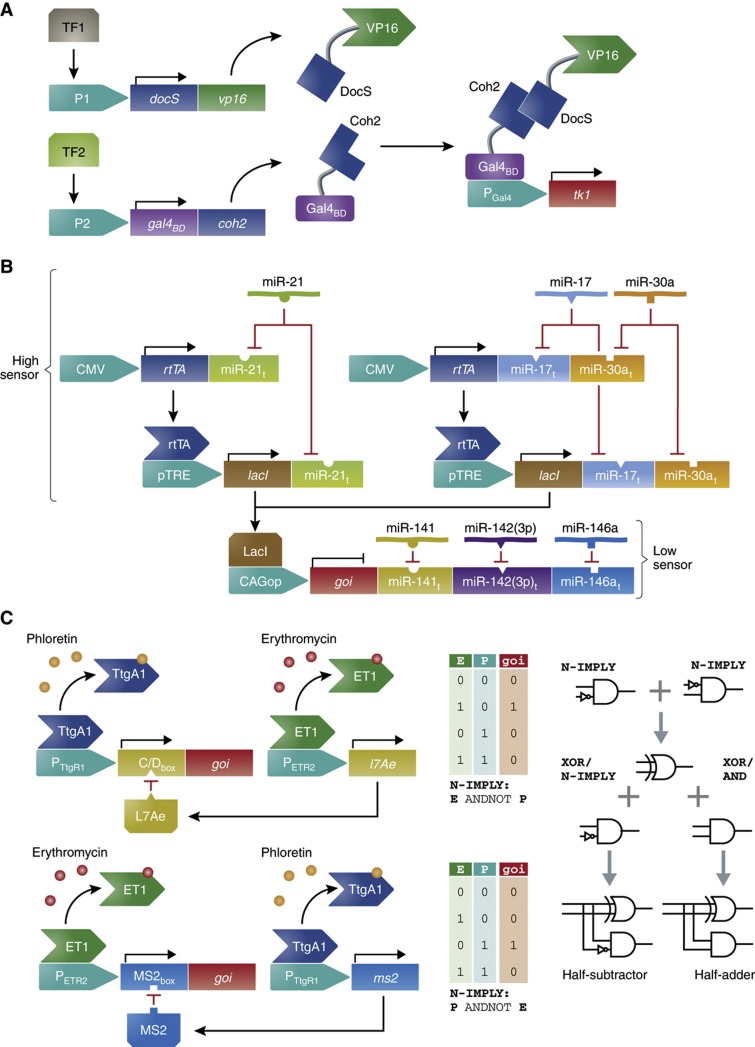
Multi-input design for increased circuit complexity. (**A**) Two-input circuit for cancer cell recognition and destruction. The synthetic promoters CXCL1, SSX1 and H2A1, which show diverse activation strengths in various cancer cell lines, are engineered to control the gene expression of either one of two subunits, DocS-VP16 and Gal4_BD_-Coh2, which together comprise a split transactivator. As the activities of the synthetic promoter combinations (P1; either CXCL1, SSX1 or H2A1, P2; either CXCL1, SSX1 or H2A1) used are regulated by endogenous, cell-specific transcription factors (TF1, TF2), the split transactivator is only expressed in a cell line where sufficient activities of both promoters are obtained. The association of DocS and Coh2 produces a functional transactivator that activates gene expression of a killer gene (TK1) from a Gal4-synthetic promoter (P_Gal4_), thus leading to cell death (Nissim and Bar-Ziv, 2010). (**B**) Multi-input circuit for cancer cell recognition and destruction. A cell type classifier for HeLa cells was constructed by implementing endogenous expressed microRNA profiles consisting of high- or low-expressed microRNA (high/low sensors). Three high-expressed microRNAs (miR-21, miR-17 and miR-30a) targeted the mRNA of the activator rtTA and the repressor LacI (miR-21_t_, miR-17_t_ and miR30a_t_). rtTA was designed to activate the expression of LacI and LacI in its turn was designed to repress the final expression of a output gene (GOI), thereby only allowing for the activation of the gene in the presence of all three high-expressed microRNAs. Three low-expressed microRNAs (miR-141, miR142(3p) and miR-146a) further targeted the mRNA of the output gene (miR-141_t_, miR-142(3p)_t_ and miR-146a_t_), only allowing for its expression at low levels of all three of the microRNAs. Regulation of a killer gene (hBax) with this cancer cell classifier enabled cell type-specific destruction of the HeLa cells (Xie et al, 2011). (**C**) Two-input circuits enable construction of plug-and-play assemblies performing sophisticated computations. The transcription factors ET1 and TtgA_1_, which repress the promoter activity of P_ETR2_ and P_TtgR1_ in response to erythromycin (E) and phloretin (P), were combined with the RNA-binding proteins MS2 and L7Ae, which inhibit the translation of transcripts containing the specific target motifs MS2_box_ and C/D_box_, to construct circuits capable of performing easy computations such as N-IMPLY logics, which are induced in the presence of only one specific input molecule. Assembling such simple circuits in a plug-and-play fashion allowed the construction of complex circuits capable of performing half-subtractor and half-adder computations (Auslander et al, 2012a).

A simple yet efficient strategy for designing two-input circuits able to respond to AND-gate logics was illustrated by Nissim and Bar-Ziv (2010). They used the activation strengths of the synthetic promoters CXCL1, SSX1 and H2A1 in various cancer cell lines. Each promoter regulated the expression of one of the two components in a split transcription factor, enabling functional gene activation only when both promoters used were sufficiently active. The split transcription factor consisted of two fusion proteins, one of which was the bacterial DocS fused to the viral VP16 transactivation domain, and the other of which was the bacterial Coh2 fused to the yeast Gal4-DNA-binding domain. DocS–Coh2 association and subsequent activation of a Gal4 synthetic promoter by the associated transcription factor were dependent on the combined activity of CXCL1, SSX1 and H2A1 promoters. As the levels of endogenous transcription factors in turn controlled the activity of these promoters, this system allowed for cancer cell-specific recognition and the production of a response modifying subsequent cancer cell fate (Figure 2A) (Nissim and Bar-Ziv, 2010).

A highly sophisticated multi-input design circuit, which allowed for specific cancer cell recognition and destruction, has been reported by Xie et al (2011). They constructed a cell-type classifier that scored high and low levels of cancer cell-specific microRNAs and when matching the predetermined profile, programmed the identified cancer cells for apoptosis. The high-level microRNA markers, miR-21, miR-17 and miR-30a, targeted mRNA of the transactivator rtTA and the transrepressor LacI. rtTA was designed to activate expression of LacI, while LacI in turn was designed to repress the expression of the apoptosis-inducing hBax, by binding to the CAGop promoter. High levels of all three high-level microRNA markers would be required for the expression of hBax. Low microRNA markers, miR-141, miR-142(3p) and miR-146a, were set to target the translation of hBax. This enabled the apoptosis-inducing transgene to be only translated if the levels of all three low-level microRNA markers are indeed low. When the cell classifier locked into the specific high- and low-level microRNA profile it executed specific destruction of matching cancer cells (Figure 2B) (Xie et al, 2011).

The possibility of designing synthetic circuits capable of performing logic gate calculations was a landmark advance in synthetic biology (Kramer et al, 2004a; Rinaudo et al, 2007). Recent work by Auslander et al (2012a) presents the engineering of combinatorial circuits, using integrated two-molecule input, capable of performing complex logic calculations. For the construction of such circuits they used the transcription factors ET1 and TtgA_1_, which respond to erythromycin and phloretin, as well as the RNA-binding proteins MS2 and L7Ae, which inhibit the translation of transcripts containing the specific RNA target motifs MS2_box_ and C/D_box_. In a plug-and-play fashion, implementing these simple transcription-translation control elements, trigger-programmable circuits able to process NOT, AND, N-AND and N-IMPLY logics were constructed. XOR computations were achieved by different combinations of two N-IMPLY gates and the combination of three logic gates enabled cells to perform calculations as complex as additions (one AND gate and two N-IMPLY gates) and subtractions (three N-IMPLY gates) (Figure 2C) (Auslander et al, 2012a).

## Light-responsive synthetic circuits

Light-sensing proteins are abundant in nature, and they permit light energy to be transferred into specific cellular responses (Sharrock, 2008; Do et al, 2009). Examples include the microbial light-sensitive ion channels called opsins, which become permeable to ion fluxes in response to light. The introduction of opsins into mammalian cells has in recent years become a powerful biological tool called optogenetics, which allows for spatiotemporal control of cellular functions (Zhang et al, 2006). When engineered to express light-responsive opsin, the activation state of single neuronal cells can be regulated (Boyden et al, 2005), heart function can be controlled (Arrenberg et al, 2010; Bruegmann et al, 2010) and vision restored (Doroudchi et al, 2011) simply by applying light. The potential of light as a non-invasive regulator of functions at the cellular, organ and even organism level has not gone unnoticed by synthetic biologists (Schroder-Lang et al, 2007; Airan et al, 2009; Chaudhury et al, 2013). Optogenetics has been shown to be a powerful tool in mammalian synthetic biology, allowing for precise and easy control of cell fate with spatiotemporal precision.

## Blue light-controlled circuits

Photosensitive proteins found in nature dimerize when exposed to light, and this property is being used to generate light-responsive synthetic circuits in mammalian cells. Such light-responsive elements include photoreactive light-oxygen-voltage domains (LOV domains) bound to the co-factor flavin mononucleotide (FMN), which upon blue-light absorption enable protein–protein interactions in prokaryotes, fungi and plants, and in doing so, regulate various cellular functions (Demarsy and Fankhauser, 2009; Herrou and Crosson, 2011).

**Figure 3:**
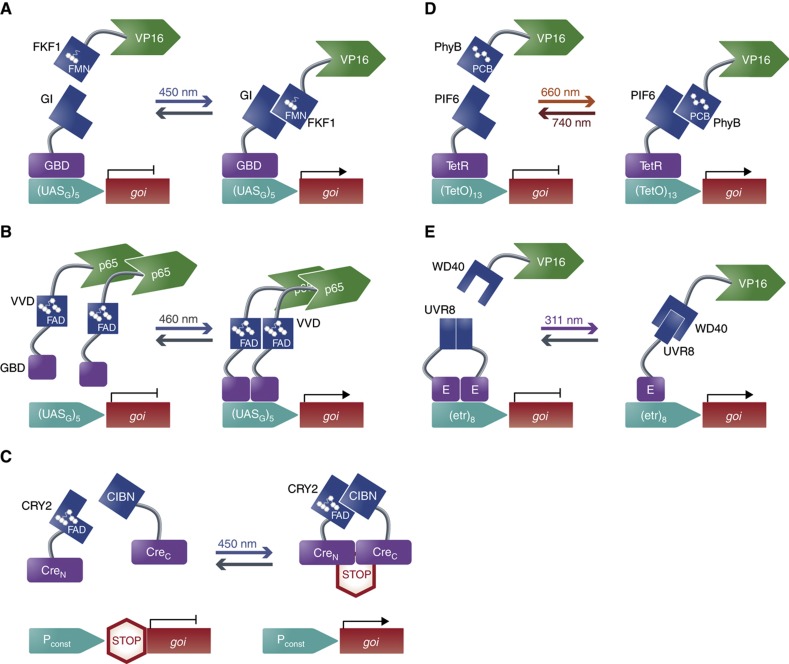
Synthetic circuits responsive to light. (**A**–**C**) Blue light-controlled circuits. (A) The two proteins GI and FKF1 with its chromophore flavin mononucleotide (FMN) interact upon blue light. Fusions of GI to the Gal4-DNA-binding domain (GBD) and FKF1 to the VP16 activation domain enable blue light-dependent association of the split transactivator, which consequently activates gene expression from a Gal4-promoter (UAS_G_)_5_ (Yazawa et al, 2009). (**B**) A fusion protein composed of VVD fused to the p65 activator and a monomeric variant of the Gal4-DNA-binding domain (GBD) is unable to bind to the Gal4 promoter ((UAS_G_)_5_) and activate gene expression due to the monomeric structure of the GBD. Blue-light illumination enables VVD dimerization due to its chromophore flavin adenine dinucleotide (FAD), thus reconstituting the GBD dimer and consequently activating gene expression (Wang et al, 2012). (**C**) Fusion proteins of CRY2 and CIBN to each part of a split Cre recombinase lacking enzymatic activity (Cre_N_ and Cre_C_) enabled associated and reconstituted Cre activity through the blue light-dependent interaction of CRY2, which requires FAD, and CIBN. The functional Cre acts by eliminating a stop sequence flanked by loxP sites, subsequently permitting gene expression (Kennedy et al, 2010). (**D**) Red light-controlled circuit. The two proteins PhyB and PIF6 interact upon red light while far-red light inhibits the interaction. Fusions of PhyB, which uses the chromophore phytochromobilin (PCB), to VP16 and PIF6 to the TetR repressor enabled red light-dependent association of the split transactivator, consequently activating gene expression from a TetR-promoter ((TetO)_13_). This action was reversed using far-red light, which caused dissociation of the PhyB and PIF6 fusions (Muller et al, 2013a). (**E**) UVB light-controlled circuit. Fusion proteins of UVR8 to the E repressor and WD40 to VP16 enabled association of the split transactivator upon UVB illumination as the UVR8 homo-dimerization is released, allowing for WD40-VP16 recruitment. The reconstituted transactivator enables gene expression from a promoter containing an E-responsive operator motif ((etr)_8_) (Muller et al, 2013b).

Implementing the blue light-dependent protein–protein interaction of the *Arabidopsis* derived flavin-binding kelch repeat f-box 1 (FKF1), containing an LOV domain, to the GIGANTEA protein (GI) resulted in the first light-regulated transgene expression system in mammalian cells (Yazawa et al, 2009). Yazawa *et al* fused GI to a Gal4-DNA-binding protein and FKF1 to a VP16 transactivation domain. Upon blue-light illumination, the FKF1-VP16 fusion protein was recruited to the GI-Gal4-DNA-binding protein, thereby enabling activation of gene expression from its cognate promoter containing Gal4-specific operator sites (Figure 3A) (Yazawa et al, 2009). Replacement of the Gal4-DNA-binding protein with a zinc finger protein (ZFP) made it possible to target specific sequences with engineered ZFP, thereby opening the possibility of also regulating endogenous genes in response to light (Polstein and Gersbach, 2012).

The smallest LOV domain-containing protein VIVID (VVD), derived from *Neurospora crassa*, incorporates the co-factor flavin adenine dinucleotide (FAD). VVD was utilized by Wang et al (2012) to engineer blue light-inducible regulation. A modified version of VVD was fused to a monomeric variant of the Gal4-DNA-binding domain and the p65 transactivation domain. Upon blue-light illumination, VVD was able to dimerize, consequently allowing the reconstituted Gal4-DNA-binding domain dimer to bind to its cognate promoter and activate gene expression. This design allowed for spatial control of gene expression in mice (Figure 3B) (Wang et al, 2012).

Blue light-induced protein–protein interaction found in *Arabidopsis thaliana*, between cryptochrome 2 (CRY2), which requires FAD as a co-factor, and the cryptochrome-interacting basic-helix-loop-helix (CIB1), was implemented to regulate transgene expression by fusing the dimerization partners to the two parts of an artificially split Cre recombinase (Kennedy et al, 2010). Blue light enabled these parts to combine and thus produce Cre activity. This eliminated a stop sequence flanked by two loxP sites, thereby allowing for gene expression (Figure 3C) (Kennedy et al, 2010). CRY2 has further been implemented in achieving blue light-mediated protein oligomerization and photoactivation of the endogenous β-catenin pathway (Bugaj et al, 2013).

Blue light has also been utilized to directly control protein function by stimulating enzymatic activity upon exposure to blue light (Wu et al, 2009; Zhou et al, 2012), achieving blue light-responsive migration of stem cells in synthetic extracellular matrices (Guo et al, 2012) and enabling blue light-guided protein localization (Strickland et al, 2012).

## Red light-controlled circuits

Not only blue light-responsive optogenetic tools have been introduced in mammalian cell-based synthetic biology. Concurrent with the first blue light-based systems came red light-based systems where precise spatiotemporal control of cellular morphology was demonstrated by a system utilizing the plant phytochrome B (PhyB) and its interaction with phytochrome interacting factor 6 (PIF6) upon exposure to red/far-red light. This system required exogenous addition of the co-factor phytochromobilin (PCB) (Levskaya et al, 2009).

Capitalizing on the interaction mechanism of PhyB and PIF6, the first mammalian gene regulation system responsive to red light was constructed (Muller et al, 2013a). Muller *et al* engineered a split transcription factor based on the fusion proteins of the tetracycline repressor TetR to PIF6 and PhyB fused to the VP16 transactivation domain. Red light enabled the reconstitution of the split transcription factor, thereby activating gene expression from a TetR-specific target promoter. Far-red light illumination resulted in the dissociation of PhyB from PIF6 and the de-activation of gene expression (Figure 3D). The authors further showed its utility by inducing spatially controlled angiogenesis in chicken embryos using red light-controlled expression of the human vascular endothelial growth factor splice variant 121 (hVEGF_121_) (Muller et al, 2013a).

## Ultraviolet B light-controlled circuits

A genetic circuit responding to ultraviolet B (UVB) light has recently been reported (Muller et al, 2013b). The authors used the *A. thaliana* photoreceptor protein UV resistance locus 8 (UVR8), which homo-dimerizes in the absence of UVB light, and the WD40 domain of its interacting partner COP1. By fusing UVR8 to the macrolide repressor E and WD40 to the VP16 transactivation domain, they constructed a split transcription factor that was activated upon exposure to UVB light, which released UVR8 homo-dimerization, allowing for WD40-VP16 recruitment. The reconstituted transcription factor then activated gene expression from a chimeric promoter containing the E-responsive operator motif (etr_8_) (Figure 3E). Finally, multichromatic control of gene expression was established by combining light control circuits responding to blue, red and UVB light. Such multichromatic systems were implemented in a circuit used to control angiogenesis signaling processes (Muller et al, 2013b).

## Engineering intercellular communication

Humans communicate via speech, but simpler organisms such as bacteria developed ways to communicate through direct exchange of molecules to monitor and adapt to their environment. For example, quorum sensing enables bacteria to synchronize activities, such as motility and gene expression, within a large group of cells, thereby adapting population-wide behavior (Bassler, 1999; Waters and Bassler, 2005). At the cellular level of the human body, specialized cells, such as those of the immune- or endocrine systems, communicate through signaling molecules to regulate crucial biological processes. The natural existence of specialized cells, performing specific tasks that are coordinated by intercellular signaling, has in recent years inspired the design of synthetic multicellular assemblies (Weber et al, 2007a; Bacchus et al, 2012; Ortiz and Endy, 2012; Rusk, 2012). Not only does synthetic intercellular communication networks represent a way for synthetic biologists to build and thereby understand naturally existing systems (Weber et al, 2007a; Balagadde et al, 2008; Song et al, 2009), but it also allows to design gene network topologies with increasing complexity and new control dynamics (Bacchus et al, 2012). Intercellular communication enables the engineering of genetic circuits that allow for robust and timely gene expression in entire cellular populations (Prindle et al, 2012), the possibility for programmed pattern formation (Basu et al, 2005; Liu et al, 2011), as well as the creation of interconnected multicellular assemblies, very similar to those found in nature (Bacchus et al, 2012; Macia et al, 2012).

**Figure 4:**
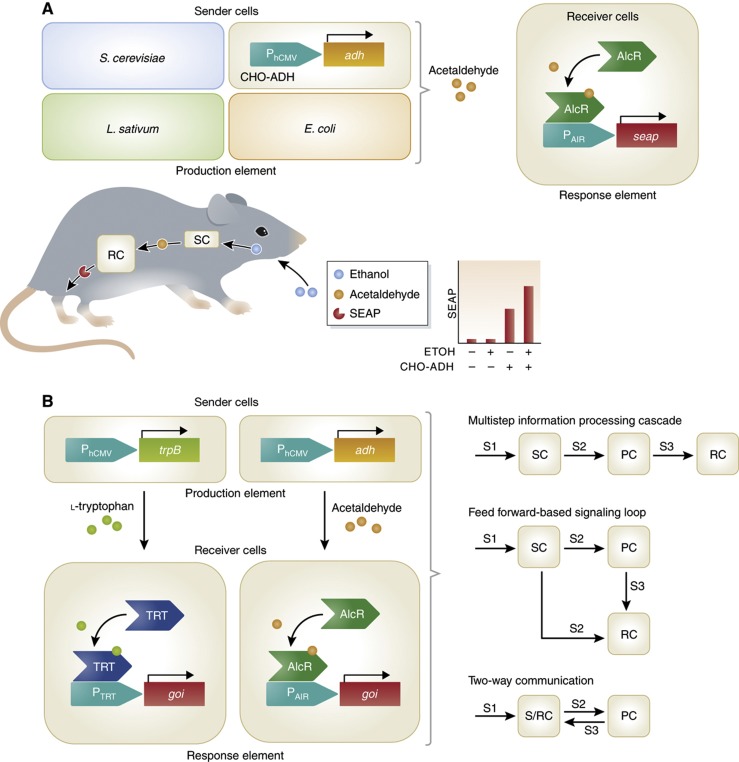
Engineering of intercellular communication. (**A**) Acetaldehyde-based intercellular communication system enables interkingdom communication. Sender cells (SCs), able to produce acetaldehyde, composed of *E. coli*, *S. cerevisiae*, *L. sativum* or mammalian cells engineered to express alcohol dehydrogenase. Mammalian receiver cells (RCs) were engineered with an acetaldehyde-responsive element consisting of AlcR, which in the presence of acetaldehyde activates gene expression from a P_AIR_ promoter. Implanting the mammalian sender and receiver cells in mice allowed for the production of acetaldehyde by the sender cells, thus converting ethanol supplemented in the drinking water. The acetaldehyde was broadcast to the receiver cells allowing for gene expression of secreted alkaline phosphatase (SEAP) (Weber et al, 2007a). (**B**) L-tryptophan-based intercellular communication system enables multicellular assemblies. Sender cells were engineered to express tryptophan synthase (TrpB), converting supplemented indole into L-tryptophan. The receiver cells were engineered with an L-tryptophan-responsive element consisting of the transactivator TRT, which activates gene expression from P_TRT_ in the presence of L-tryptophan. Combining the genetic components of the acetaldehyde- and L-tryptophan-based intercellular communication systems allowed for various sender- (SC), processor- (PC), receiver (RC) and sender/receiver cells (S/RC) to be constructed. Assembling these components in a plug-and-play manner allowed the creation of multicellular architectures mimicking the natural phenomena (Bacchus et al, 2012).

The first synthetic intercellular communication system in mammalian cells, constructed by Weber et al (2007a), allowed engineered sender cells to produce a metabolic signal in a cell density-dependent manner, and engineered receiver cells to respond to that signal with a distinct genetic response. The sender cells were engineered to express mouse-derived alcohol dehydrogenase (ADH), allowing supplemented ethanol to be converted into the volatile metabolite acetaldehyde. The receiver cells were engineered with an acetaldehyde-inducible regulation system based on the genetic components derived from *Aspergillus nidulans*, which enabled gene expression upon reception of acetaldehyde. Replacement of the engineered mammalian sender cells with those of *E. coli*, *S. cerevisiae* and *L. sativum*, organisms naturally expressing ADH, allowed for interkingdom communication, as the produced acetaldehyde was routed to the mammalian receiver cells. When microencapsulated circuit-transgenic designer cells were implanted into mice, the mammalian sender and receiver cells functioned in a manner similar to hormones. Ethanol provided through drinking water was converted by the sender cells into acetaldehyde and broadcast to the receiver cells, thereby triggering transgene expression (Figure 4A) (Weber et al, 2007a).

The generic design for constructing intercellular communication in mammalian cells (Weber and Fussenegger, 2011) by implementing distinct sender and receiver cell populations has been adapted to create intercellular communication systems responding to L-arginine (Weber et al, 2009), biotin (Weber et al, 2007a), nitric oxide (Wang et al, 2008) and L-tryptophan (Bacchus et al, 2012). The latter system was composed of sender cells engineered to express the bacterial gene tryptophan synthase (TrpB), allowing for the conversion of supplemented indole into the amino acid L-tryptophan. The receiver cells expressed a target gene via a constructed L-tryptophan-inducible regulation system based on the genetic components derived from *Chlamydia trachomatis.* The potential of intercellular communication in bioreactor settings, which could be important in manufacturing pharmaceuticals or biofuels, was illustrated by programming gene expression profiles to be dependent on inoculated cell concentrations. Combining the acetaldehyde and L-tryptophan intercellular communication systems allowed for complex multicellular assemblies to be constructed. Natural signaling systems of multicellular assemblies such as multistep information processing cascades, feed forward-based signaling loops, and two-way communication were mimicked simply by implementing the same genetic building blocks in different cellular configurations (Figure 4B). For example, two-way communication was used in a model for angiogenesis by controlling vascular endothelial cell permeability (Bacchus et al, 2012).

## Prosthetic networks

Prosthetic networks are synthetic devices which will act as molecular prosthesis that sense, monitor and score (disease-) relevant metabolites, process off-level concentrations and coordinate adjusted diagnostic, preventive or therapeutic responses in a seamless, automatic and self-sufficient manner (Weber and Fussenegger, 2012; Auslander and Fussenegger, 2013; Perkel, 2013). In contrast to the aforementioned transgene control devices, prosthetic networks are directly linked to host metabolism and triggered by the disease metabolite.

**Figure 5:**
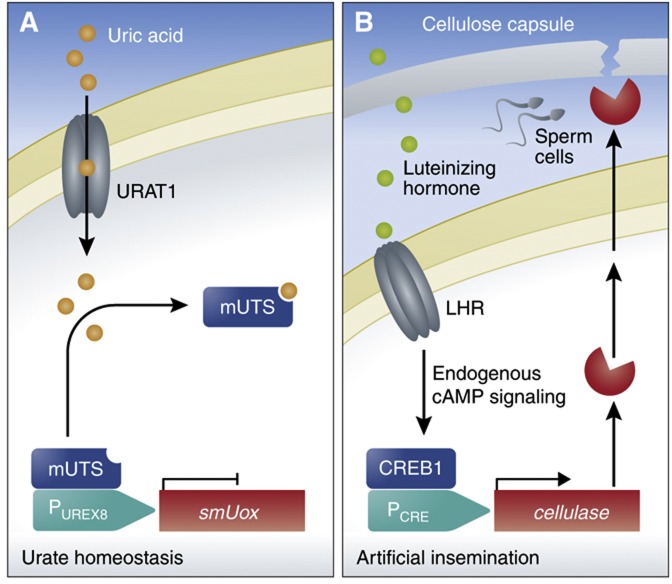
Prosthetic networks. (**A**) Prosthetic network regulating urate homeostasis. Cells are engineered to respond to elevated levels of uric acid by disassociation of the mUTS repressor from the P_UREX8_ promoter, thereby enabling transgene expression. Expression of a urate transporter (URAT1) enhanced intercellular urate concentrations and circuit sensitivity. When implanted in urate oxidase-deficient mice, the circuit sensed pathologically high levels of uric acid in the blood stream, activated transgene expression of a secreted urate oxidase (smUox), and thus reduced the elevated levels of uric acid (Kemmer et al, 2010). (**B**) Prosthetic network for artificial insemination. Cells engineered to express the luteinizing hormone receptor (LHR) respond to luteinizing hormone by activating endogenous cAMP signaling, allowing for the activation of P_CRE_-driven transgene expression of cellulase via a cAMP-responsive element binding protein 1 (CREB1). Engineered cells are co-encapsulated with sperm into cellulose-based implants and positioned in the uterus of cows. Ovulation-coordinated activation of cellulase expression in response to elevated levels of luteinizing hormone results in capsule degradation and sperm release (Kemmer et al, 2011).

The potential to use prosthetic networks as therapy was demonstrated in a pioneering example reported by Kemmer et al (2010). Elevated levels of uric acid are associated with pathological conditions such as tumor lysis syndrome and gout, so they constructed a genetic circuit for controlling uric acid homeostasis in mice. The circuit was composed of a modified *Deinococcus radiodurans*-derived protein (mUTS) able to relieve repression of its cognate promoter (P_UREX8_) upon elevated levels of uric acid. After insulation of circuit-transgenic cells by encapsulation in immunoprotective microcontainers (Auslander et al, 2012b) and implantation in urate oxidase-deficient mice developing gout, the circuit autoconnected to peripheral circulation, sensed the pathologically high levels of uric acid in the bloodstream of the animals, activated the expression of a secretion-engineered version (smUox) of the clinically licensed *Aspergillus flavus* urate oxidase (Rasburicase) driven by P_UREX8_, and thereby reduced the levels of uric acid to subpathological levels (Figure 5A) (Kemmer et al, 2010).

Prosthetic networks have also been developed as a tool for artificial insemination (Kemmer et al, 2011). This was achieved by rewiring the luteinizing hormone receptor (LHR) to activate a CREB1, enabling transgene expression when the receptor was stimulated by the luteinizing hormone binding to it. The stimulation of LHR triggered a classic G protein-coupled receptor response, which increased levels of intracellular cAMP, and triggered CREB1 binding to a synthetic promoter (P_CRE_) controlling expression of a secretion-engineered cellulase. Co-encapsulation of sperm and cells containing this circuit into cellulose-based capsules were implanted in the uterus of cows. At ovulation, elevated levels of luteinizing hormone were produced, which resulted in the rupture of the implants when the secreted cellulase degrades the cellulose-based capsule, and ultimately resulted in successful fertilization (Figure 5B) (Kemmer et al, 2011).

## Conclusion

Starting from the basic construction of transcriptional gene regulation systems, and utilizing native bacterial gene switches that respond to antibiotics, synthetic biology circuits today include novel circuits based on the transcriptional, translational or post-translational regulation (Auslander and Fussenegger, 2013). These complex circuits are designed for use in therapy, as they are engineered to respond to metabolites of the human body, to native cell-signaling pathways and to disease or cell-specific markers and thus target specific disease states (Wei et al, 2012). This development is by no means coincidental, as researchers have worked to find synthetic biology solutions for real clinical issues (Dean et al, 2009; Ruder et al, 2011; Folcher and Fussenegger, 2012; Weber and Fussenegger, 2012).

Yet, can synthetic biology deliver what it promises outside the laboratory as well? To achieve these ambitious goals, it is crucially important to solidify the advances that have been made in standardized genetic circuit design, and to create still more robust and complex circuits, as these in turn will ensure safe and reliable usage (Endy, 2005; Gardner, 2013). Capitalizing on the most recent technological advances in synthetic biology, time has now come that these designer devices be implemented and validated in clinical settings. Therefore, the designer cells will have to traverse the same clinical phases and likely meet with similar technical challenges as gene- and cell-based therapies. However, with over two decades of records in gene-based treatment strategies, clinical implementation of synthetic biology devices may be more straightforward (de Amorim, 2009; Laurencot and Ruppel, 2009; Deplazes-Zemp and Biller-Andorno, 2012; Philp et al, 2013).

In just a few years, optogenetics has become of marked importance for synthetic biology (Knopfel et al, 2010; Chow and Boyden, 2011). If applied in clinical settings, then the regulation of crucial therapeutic proteins in response to light could be a reality for patients. This would provide an optimal solution to biopharmaceutical production, as such therapy could be used to induce protein production at a specific cell density without the addition of chemical inducers (Ye et al, 2011). While most mammalian light circuits are controlled by blue light, red light systems could prove to be highly influential, as red light penetrates tissue more efficiently than blue light does (Muller et al, 2013a). However, the clinical utility of light-controlled circuits is limited by their chromophores. For example, the red light-controlled circuit requires phytochromobilin, which is not only difficult to produce and to administer but also unlikely to become clinically licensed due to side effects caused by this plant-derived co-factor. Also, light-controlled devices assembled from human components are preferred to eliminate the risk of immune responses and other undesired site effects. With its all-human design and the ubiquitous co-factor vitamin A, the blue light-responsive melanopsin-derived optogenetic device meets the high standard clinical compatibility (Ye et al, 2011) (Figure 1B). The development of multichromatic control circuits will further broaden the biomedical utility of light-controlled circuits and enable more accuracy in the implementation of the circuits (Muller et al, 2013b).

With the introduction of synthetic intracellular communication systems, synthetic biologists have not only found an innovative way to tackle the current processing limitations of single cells, but have also found a solution to design the circuits of the future which likely continue to increase in complexity and thus require more components (Perkel, 2013). As intercellular communication allows for spatial separation of the cell populations, it could hold great promise for biomedical applications such as advanced tissue engineering. Implementation of multiple and interconnected cell implants *in vivo* could allow for remote control of differing functions, very much like the natural regulatory processes in the body. The application of engineered intercellular communication systems for therapeutic purposes is not only restricted to mammalian cell design (Anderson et al, 2006; Duan and March, 2010; Wu et al, 2013). With their cell density-dependent transgene expression responses, intercellular communication systems represent a powerful asset for synthetic biology (Mitchell et al, 2011; Miller et al, 2012; Shong et al, 2012).

Increased complexity, reliability and accuracy of genetic circuit devices in combination with incorporating newly developed technologies will ensure synthetic biology's place among the biological engineering disciplines of the 21st century. This century is likely to mark mammalian synthetic biology's advance from a ‘proof of concept' discipline to a tool commonly used in clinical medical practice.

## Figures and Tables

**Figure:**